# Disease activity, resilience and health-related quality of life in Chinese patients with rheumatoid arthritis: a multi-center, cross-sectional study

**DOI:** 10.1186/s12955-017-0725-6

**Published:** 2017-07-24

**Authors:** Li Liu, Xin Xu, Neili Xu, Lie Wang

**Affiliations:** 1 Department of Social Medicine, School of Public Health, China Medical University, No.77 Puhe Road, Shenyang North New Area, Shenyang, Liaoning 110122 People’s Republic of China; 2 Department of Clinical Epidemiology, Shengjing Hospital of China Medical University, No.36 Sanhao Street, Heping District, Shenyang, Liaoning 110004 People’s Republic of China; 3 Department of Rheumatology, Shengjing Hospital of China Medical University, No.36 Sanhao Street, Heping District, Shenyang, Liaoning 110004 People’s Republic of China

**Keywords:** Health-related quality of life, SF-36, DAS28-CRP, Positive psychology, Rheumatoid arthritis

## Abstract

**Background:**

Positive psychological constructs that can moderate or mediate the negative impact of disease activity on health-related quality of life (HRQOL) in patients with rheumatoid arthritis (RA) have not been explored widely. This study aimed to assess the associations of disease activity, resilience with HRQOL and the moderating and mediating roles of resilience among Chinese RA patients.

**Methods:**

A multi-center, cross-sectional study was conducted in RA inpatients in northeast of China. A total 298 subjects completed the Medical Outcomes Study 36-item Short-Form Health Survey (SF-36) and Ego-Resiliency Scale (ERS) to measure HRQOL and resilience. For the SF-36, physical function, physical role limitation, bodily pain and general health perception are gathered into physical component summary (PCS), while vitality, social functioning, emotional role limitation and mental health are gathered into mental component summary (MCS). Disease activity was evaluated by the Disease Activity Score 28-C-reactive protein (DAS28-CRP). Hierarchical regression analysis was applied to examine the associations of disease activity, resilience and the disease activity*resilience interaction with PCS and MCS, respectively. Asymptotic and resampling strategies were utilized to examine the mediating role of resilience.

**Results:**

The mean scores of PCS and MCS were 40.67 and 59.14, respectively. Disease activity was negatively associated with both PCS and MCS, and resilience was only positively associated with MCS. The disease activity*resilience interaction term were significantly associated with MCS (*β* = 0.144, *P* = 0.003). The associations between disease activity and MCS were gradually reduced in low (1 SD below the mean, *β* = −0.369, *P* < 0.001), mean (*β* = −0.218, *P* < 0.001) and high (1 SD above the mean, *β* = −0.068, *P* = 0.369) groups of resilience. Resilience acted as a partial mediator in the disease activity-MCS association (effect size was −0.085, BCa 95% *CI*: −0.159, −0.028).

**Conclusions:**

Disease activity was negatively associated with both physical and mental HRQOL, and resilience was only positively associated with mental HRQOL. Resilience could attenuate and mediate the association between disease activity and mental HRQOL. In addition to controlling disease activity, targeted intervention strategies designed for resilience should be strengthened to improve the HRQOL of this population.

## Background

Rheumatoid arthritis (RA) is a chronic, symmetric and progressive inflammatory disease that includes some disease-specific symptoms such as joint pain, stiffness, swelling and fatigue [1]. RA can lead to disability and significantly interfere with functional adaptation [1]. RA-physical and functional stressors together create enormous psychological distress. RA patients often have a high prevalence of emotional stress, depression and anxiety [2]. Thus, considerable physical and psychological challenges can seriously damage these patients’ overall health-related quality of life (HRQOL) [3, 4]. In general, HRQOL refers to the ways in which a given health condition affects a patient’s physical ability and capacity to function in various social and emotional roles, including physical functioning and mental well-being [5].

Certainly, it is important to identify factors that can influence HRQOL to make it a more feasible target for clinical management. According to the Wilson and Cleary model (WCM) [6], six categories are directly or indirectly related to patients’ overall HRQOL: biological and physiological status, symptom status, functional status, general health perceptions, individual characteristics, and environmental characteristics. In the WCM, a causal pathway between biological and physiological status and HRQOL is proposed through sequential effects on symptoms, functions and general health perceptions [6]. Therefore, biological and physiological status can play prominent roles in explaining physical functioning and mental well-being in RA patients [7, 8].

Disease activity is one of the indicators of the clinical assessment of biological and physiological status to guide treatment adjustment in RA [9]. Previous qualitative studies have shown a strong relationship between disease activity and HRQOL including physical and mental components [7, 8, 10, 11]. In addition, a common observation has been reported that there is a significant variability in health functioning among RA patients who have similar levels of disease activity [10, 11]. The finding gives a strong hint that there are some individual and environmental factors that play a role in mediating or moderating the relationship between disease activity and HRQOL in RA patients [10]. Therefore, those potential mediators and moderators are necessary to be identified for improving HRQOL promptly through targeted intervention, while trying to reduce the disease activity of RA. However, the contribution of such factors to HRQOL has not been adequately determined in RA patients.

With the rise of positive psychology, researchers have gradually shifted attention to the exploration of the effects of positive psychological constructs on health outcomes in patients with chronic diseases [12, 13]. Positive psychological capacities seem not only to be associated with good physical and mental outcomes in those patients, but also to play a vital role in re-realizing their conditions [14]. In clinical practice, several interventions integrating positive psychological capacities have shown encouraging health outcomes [15, 16]. Among these positive psychological constructs, resilience is a focus in this field. With regard to definition, resilience generally refers to the dynamic capacity of someone to bounce back from life adversities to maintain or recuperate his health successfully [17]. Rutter proposed four possible mechanisms of resilience: (1) reducing the impact of risk factors, including changes in individuals’ cognition about risk factors, and avoiding or reducing contact with them; (2) reducing the negative chain reaction of negative life events; (3) improving the levels of self-esteem and self-efficacy; (4) helping individuals access available resources for hope and success [17]. In view of the results of previous studies, resilience can directly indicate lower levels of perceived distress, better adjustment and health outcomes among patients with chronic diseases, such as RA, cancer, diabetes and psychiatric disorders [18–21]. Moreover, resilience can also act as a ‘moderator’ to enhance or attenuate the effects of stressors on physical and mental well-being. Rainone et al. found that there was a moderating role of resilience on the relationship between affective disorders and HRQOL for adolescents and young adults with multiple sclerosis [22]. Min et al. reported that resilience played moderation on the negative effect of pain on depression and post-traumatic growth in individuals with spinal cord injury [23]. In addition, previous researches indicated that resilience played a mediating role in the relationship between cancer symptom distress and HRQOL [24, 25]. Thus, resilience probably could help explain individual variation in HRQOL in patients with chronic diseases. However, the positive role of resilience on HRQOL, and whether or not resilience moderates or mediates the relationship between disease-related physiological status and HRQOL have not been examined among Chinese RA patients to our best knowledge.

In light of the above concerns, the present study aimed to assess the effects of disease activity and resilience on HRQOL, and to examine the moderating and mediating roles of resilience on the association between disease activity and HRQOL in Chinese RA patients.

## Methods

### Ethics statement

The study was approved by the Ethics Committee on Human Experimentation of Shengjing Hospital of China Medical University, Central Hospital of Benxi, General Hospital of Fushun Mining Bureau and Sujiatun Central Hospital of Shenyang, and was conducted in accordance with the principles contained in the Declaration of Helsinki for studies in humans. All the patients provided their written informed consent after being informed about the objectives and procedures of the study, and they participated in the study voluntarily and anonymously.

### Study design and sample

This was a multi-center and cross-sectional study. From December 2014 through January 2016, successive inpatients diagnosed with RA were recruited at the Department of Rheumatology at Shengjing Hospital of China Medical University, Central Hospital of Benxi, General Hospital of Fushun Mining Bureau and Sujiatun Central Hospital of Shenyang. The four medical centers gather a substantial numbers of patients in Liaoning Province that is in the northeast of China.

In this study, the inclusion criteria of patient recruitment included: (1) was diagnosed with RA according to the ACR/EULAR 2010 classification criteria; (2) was at least 18 years old or older; (3) could understand and communicate in Chinese well. The exclusion criteria of patient recruitment were: (1) had a psychiatric history in the past; (2) had intellectual and/or cognitive impairments; (3) was suffering from other severe comorbidities simultaneously. Rheumatologists-in-charge requested all eligible patients to participate in the study. A set of self-administered questionnaires was distributed to the participants after obtaining the patients’ written consent. Clinical information was gathered from medical record.

In total, 325 patients were recruited as potential subjects. Twelve patients refused to cooperate subsequently. Thus, the questionnaires were distributed to 313 eligible patients, and 15 responses with missing data concerning any item in the questionnaires were excluded from the final analysis. In the final, effective responses were received from 298 patients (91.7%).

### Measures

#### Measurement of HRQOL

HRQOL was measured with the Chinese version of the Medical Outcomes Study 36-item Short-Form Health Survey (SF-36), consisting of 36 items that measured eight dimensions: physical function, role limitations related to physical problems, bodily pain, general health perception, vitality, social functioning, role limitations due to emotional problems, and mental health [5]. These dimensions were gathered into physical component summary (PCS) and mental component summary (MCS), respectively. The raw score of each component summary is converted to a standard score ranging from 0 to 100, with a higher score indicating better HRQOL perception. The SF-36 has good reliability and validity among various Chinese patients [19, 26]. In this study, the Cronbach’s α values for the PCS and MCS subscales were 0.89 and 0.88, respectively.

#### Measurement of disease activity

The disease activity of RA was indicated by the Disease Activity Score 28-C-reactive protein (DAS28-CRP) [27]. On the basis of swollen joint counts (SJC), tender joint counts (TJC) and the level of CRP, DAS28-CRP was calculated using the formula: DAS28-CRP = [0.56*sqrt(TJC28) + 0.28*sqrt(SJC28) + 0.36*ln(CRP + 1)]*1.10 + 1.15. Also, disease activity was divided into four groups based on the cut-off points of DAS28-CRP: clinical remission < 2.6, low level 2.6–3.2, moderate level 3.2–5.1, and high level > 5.1.

#### Measurement of resilience

Resilience was measured by the Chinese version of Ego-Resiliency Scale (ERS) [28], which consists of 14 items as a one-dimensional scale. The ERS emphasizes individual’s psychological capacity to bounce back especially experiencing overwhelming or stressful situations. Each item is scored on a 4-point Likert scale in accordance with the patients’ personal feelings about resilience, ranging from 1 ‘does not apply at all’ to 4 ‘completely apply’. The summed score ranges from 14 to 56, which indicates the higher the score is, the more swiftly recovery individuals make from those negative situations. The ERS fits for a variety of patients with high validity and reliability in China [29, 30], and the Cronbach’s α value for the ERS was 0.88 in this study.

#### Demographic characteristics

Demographic characteristics included gender, age, marital status, educational level, employment, household monthly income and residence. Marital status was categorized as married/cohabited and single/divorced/widowed/separated. Educational level was classified as junior high school or below, senior high school and junior college or above. Employment was sorted into two groups as unemployment and part time/full time. Household monthly income (RMB) was categorized as < 3000, < 4000 and ≥ 4000 yuan. Residence was divided into urban and rural.

#### Clinical variables

Clinical variables including TJC, SJC, CRP, duration of suffering from RA, anemia, early morning stiffness (EMS), and chronic comorbidities were collected. The duration of suffering RA was classified as: ≤ 1, 2–5, 6–10 and > 10 years. Anemia was categorized as yes or no. EMS was divided into three groups: no, ≤ 1 and > 1 h. If the respondents were suffering from other severe comorbidities, such as osteoarthritis, sclerosis, hypertension, coronary heart disease and diabetes, chronic comorbidity was defined as yes; otherwise, it was defined as no.

### Statistical analysis

All variables were described with number (n), percentage (%), mean, standard deviation (SD) and range appropriately. Group differences of continuous variables were examined using independent sample t-test or one way analysis of variance. Correlations among continuous variables were tested using Pearson’s correlation analysis. Hierarchical regression analysis was adopted to examine the associations of disease activity and resilience with HRQOL, as well as to explore the moderating role of resilience on the association of disease activity with HRQOL. Besides gender and age, demographic and clinical variables related to HRQOL in univariate analysis (*P* < 0.15) were adjusted. In step 1, gender, age, and potential control variables were added. Disease activity was added in step 2, and resilience was added in step 3. Ultimately, a disease activity*resilience interaction term was added in step 4. If the interaction effect was statistically significant, simple slope analyses were conducted to visualize the interaction term [31]. The variables in the models were centered before regression analysis. Asymptotic and resampling strategies were used to examine the mediating role of resilience in the association between disease activity and HRQOL. Bias-corrected and accelerated 95% confidence intervals (BCa 95% CI) for mediation were conducted with the bootstrap estimate on a basis of 5000 bootstrap samples, in which exclusion of 0 indicated a significant mediating role [31]. Statistical analysis was executed by SPSS 19.0 software and a two-tailed *P* < 0.05 was viewed as statistically significant.

## Results

### Descriptive statistics

Demographic and clinical characteristics and group differences in the PCS and MCS of HRQOL are displayed in Table 1. Of these subjects, 77.2% (230) were women, 88.9% (265) were married/cohabited, and they reported higher MCS scores (*t* = 2.324, *P* = 0.021) than those single/divorced/widowed/separated subjects. There were 67.1% (200) subjects received a senior high school or above education, 61.1% (182) were unemployment, 228 (76.5%) had a household monthly income level of < 4000 yuan RMB, and 217 (72.8%) lived in urban areas. With regard to clinical variables, 30 (10.1%) subjects’ DAS28-CRP scores were below 2.6, 89 (29.9%) had a RA duration of ≤ 1 year, 120 (40.3%) had anemia, 214 (71.8%) suffered from EMS, and 190 (63.8%) had at least one other chronic disease. DAS28-CRP and EMS were significantly related to both PCS and MCS, and patients with anemia reported lower PCS than those without anemia (*t* = 3.422, *P* = 0.001). However, statistically significant relations between PCS and MCS and the other demographic and clinical variables were not found in this study.

**Table 1:** Demographic and clinical variables of participants in relation to the PCS and MCS of HRQOL

Variables	*n*	%	PCS		*F/t*	*P*	MCS		*F/t*	*P*
			Mean	SD			Mean	SD		
Gender					0.626	0.532			0.621	0.549
Men	68	22.8	41.40	11.49			59.82	10.18		
Women	230	77.2	40.46	10.68			58.94	10.26		
Marital status					0.338	0.736			2.324	0.021
Single/divorced/widowed/separated	33	11.1	41.28	11.27			55.27	11.15		
Married/cohabited	265	88.9	40.60	10.82			59.62	10.03		
Educational level					0.903	0.407			0.054	0.948
Junior high school or below	98	32.9	39.74	10.20			59.21	9.34		
Senior high school	135	45.3	40.68	10.86			59.27	10.34		
Junior college or above	65	21.8	42.07	11.78			58.78	11.38		
Employment					0.418	0.676			1.312	0.191
Unemployment	182	61.1	40.46	10.27			58.52	10.21		
Part-time/full-time	116	38.9	41.00	11.75			60.11	10.24		
Household monthly income (yuan)					1.880	0.154			1.045	0.353
<3000	107	35.9	39.14	10.68			60.25	9.99		
< 4000	121	40.6	41.91	9.74			58.73	10.26		
≥ 4000	70	23.5	40.89	12.67			58.17	10.53		
Residence					1.806	0.072			0.016	0.987
Urban	217	72.8	41.36	10.52			59.15	10.42		
Rural	81	27.2	38.82	11.58			59.13	9.77		
DAS28-CRP					11.778	< 0.001			6.067	0.001
< 2.6	30	10.1	47.79	10.82			63.96	9.97		
2.6–3.2	26	8.7	42.99	9.88			61.25	9.92		
3.2–5.1	142	47.7	41.82	10.52			59.88	10.13		
> 5.1	100	33.6	36.31	9.96			56.10	9.78		
Duration (years)					0.465	0.707			0.644	0.587
≤ 1	89	29.9	41.76	12.19			59.45	11.77		
2–5	82	27.5	39.97	10.26			58.37	9.74		
6–10	52	17.4	40.08	10.53			58.19	9.43		
> 10	75	25.2	40.56	10.12			60.29	9.35		
Anemia					3.422	0.001			1.493	0.137
Yes	120	40.3	38.10	10.03			58.07	9.44		
No	178	59.7	42.41	11.07			59.87	10.70		
EMS (hours)					3.421	0.034			5.841	0.003
No	84	28.2	42.66	10.93			59.15	10.56		
≤ 1	164	55.0	40.58	10.58			60.44	9.66		
> 1	50	16.8	37.64	11.09			54.88	10.53		
Chronic comorbidity					0.236	0.814			0.964	0.336
No	108	36.2	40.87	11.61			58.38	10.81		
Yes	190	63.8	40.56	10.43			59.57	9.89		

Abbreviations: *PCS*, physical component summary; *MCS*, mental component summary; *HRQOL*, health-related quality of life; *SD*, standard deviation; *DAS28-CRP*, Disease Activity Score 28-C-reactive protein; *EMS*, early morning stiffness

The levels of PCS, MCS, age, DAS28-CRP, resilience are provided in Table 2. The mean scores of PCS and MCS were 40.67 (SD = 10.86) and 59.14 (SD = 10.23), which ranged from 7.78 to 75.35 and from 29.06 to 83.75, respectively. The mean values were 57.27 (SD = 12.11), 4.57 (SD = 1.52) and 34.22 (SD = 5.50) for age, DAS28-CRP and resilience, respectively.

**Table 2:** Descriptive statistics for continuous variables

Variables	Mean	SD	Range
PCS	40.67	10.86	7.78–75.35
MCS	59.14	10.23	29.06–83.75
Age (years)	57.27	12.11	21–82
DAS28-CRP	4.57	1.52	1.56–8.13
Resilience	34.22	5.50	23–56

Abbreviations: *SD*, standard deviation; *PCS*, physical component summary; *MCS*, mental component summary; *DAS28-CRP*, Disease Activity Score 28-C-reactive protein

### Correlations among continuous variables

The correlations among age, DAS28-CRP, resilience, PCS and MCS are displayed in Table 3. Both age and DAS28-CRP were negatively correlated with PCS and MCS, respectively. Resilience was positively correlated with MCS (*r* = 0.530, *P* < 0.01), and DAS28-CRP was negatively correlated with resilience (*r* = −0.136, *P* < 0.05).

**Table 3:** Correlations among continuous variables

Variables	1	2	3	4
1. Age	1			
2. DAS28-CRP	0.103	1		
3. Resilience	−0.101	−0.136*	1	
4. PCS	−0.129*	−0.398**	0.054	1
5. MCS	−0.140*	−0.294**	0.530**	0.587**

*, *P* < 0.05; **, *P* < 0.01. Abbreviations: *DAS28-CRP*, Disease Activity Score 28-C-reactive protein; *PCS*, physical component summary; *MCS*, mental component summary

### Hierarchical regression analysis

The results of hierarchical regression analysis on PCS are displayed in Table 4. In step 1, the linear combination of demographic and medical control variables (gender, age, marital status, residence, anemia and EMS) significantly predicted PCS (*F* = 4.128, adjusted *R* ^*2*^ = 0.069, *P* < 0.01). DAS28-CRP was significantly and negatively associated with PCS in step 2 (*β* = −0.374, *P* < 0.001), and improved model fit (*F* = 8.781, adjusted *R* ^*2*^ = 0.173, *ΔR* ^*2*^ = 0.105, *P* < 0.01). In step 3, DAS28-CRP was also significantly and negatively associated with PCS (*β* = −0.377, *P* < 0.001), whereas resilience was not significantly associated with PCS (*β* = −0.016, *P* = 0.773). In step 4, the disease activity*resilience interaction term was not significantly associated with PCS (*β* = 0.078, *P* = 0.155). Then, the statistical power of the analysis was determined using post-hoc statistical power analysis on the basis of the sample size and the observed *R* ^*2*^, assuming α = 0.05. It indicated a statistical power of 99.99% for the present sample.

**Table 4:** Hierarchical regression for exploring the correlates of PCS

Variables	Step 1		Step 2		Step 3		Step 4	
	*β*	*P*	*β*	*P*	*β*	*P*	*β*	*P*
Gender	−0.081	0.156	−0.092	0.089	−0.093	0.086	−0.101	0.062
Age	−0.166	0.005	−0.118	0.036	−0.119	0.035	−0.129	0.023
Marital status	−0.011	0.843	−0.004	0.938	−0.002	0.971	−0.001	0.991
Residence	−0.121	0.039	−0.095	0.085	−0.094	0.088	−0.094	0.089
Anemia	0.177	0.002	0.113	0.040	0.113	0.040	0.117	0.034
EMS								
Dummy_1	−0.079	0.228	0.059	0.370	0.061	0.356	0.053	0.428
Dummy_2	−0.145	0.027	0.028	0.677	0.029	0.668	0.028	0.677
DAS28-CRP			−0.374	< 0.001	−0.377	< 0.001	−0.375	< 0.001
Resilience					−0.016	0.773	−0.004	0.949
DAS28-CRP*Resilience							0.078	0.155
*F*	4.128		8.781		7.790		7.239	
Adjusted *R* ^*2*^	0.069		0.173		0.171		0.174	
*ΔR* ^*2*^			0.105		< 0.001		0.006	

Gender, women versus men; Marital status, married/cohabited versus single/divorced/widowed/separated; Residence, rural versus urban; Anemia, no versus yes; Dummy_1, EMS ≤ 1 versus no; Dummy_2, EMS > 1 versus no. * Indicated multiplication. Abbreviations: *PCS*, physical component summary; *EMS*, early morning stiffness; *DAS28-CRP*, Disease Activity Score 28-C-reactive protein

**Fig. 1:**
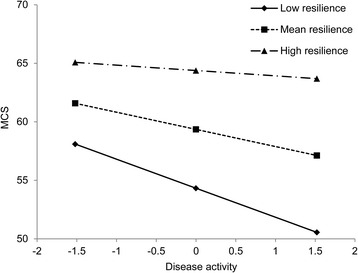
Simple slope plot of interaction between disease activity and resilience on MCS. Low, 1 SD below the mean; High, 1 SD above the mean. The values of disease activity and resilience were centered before regression analysis. The range of the centered value of disease activity is displayed. The mean and SD of the centered value of disease activity were 0 and 1.52, respectively. Gender, age, marital status, residence, anemia and EMS were adjusted. Abbreviations: *MCS*, mental component summary; *SD*, standard deviation; *EMS*, early morning stiffness

The results of hierarchical regression analysis on MCS are displayed in Table 5. In step 1, the linear combination of demographic and medical control variables also significantly predicted MCS (*F* = 3.849, adjusted *R* ^*2*^ = 0.063, *P* < 0.01). DAS28-CRP was significantly and negatively associated with MCS in step 2 (*β* = −0.308, *P* < 0.001), and improved model fit (*F* = 6.674, adjusted *R* ^*2*^ = 0.133, *ΔR* ^*2*^ = 0.071, *P* < 0.01). In step 3, DAS28-CRP was also significantly and negatively associated with MCS (*β* = −0.223, *P* < 0.001), whereas resilience was significantly and positively associated with MCS (*β* = 0.469, *P* < 0.001) and explained an additional 20.6% of the variance (*F* = 18.115, adjusted *R* ^*2*^ = 0.342, *ΔR* ^*2*^ = 0.206, *P* < 0.01). In step 4, the disease activity*resilience interaction term was significantly and positively associated with MCS (*β* = 0.144, *P* = 0.003), and explained an additional 1.9% of the variance (*F* = 17.647, adjusted *R* ^*2*^ = 0.359, Δ*R* ^*2*^ = 0.019, *P* < 0.01). Simple slope analysis revealed that when resilience was higher, the association between disease activity and MCS became weaker. In other words, the association between disease activity and MCS was gradually reduced in the low (1 SD below the mean, *β* = −0.369, *P* < 0.001), mean (*β* = −0.218, *P* < 0.001) and high (1 SD above the mean, *β* = −0.068, *P* = 0.369) groups of resilience. The interaction is visualized in Fig. 1. Furthermore, a reduced β coefficient of disease activity in step 3 suggested that resilience could mediate the disease activity-MCS association.

**Table 5:** Hierarchical regression for exploring the correlates of MCS

Variables	Step 1		Step 2		Step 3		Step 4	
	*β*	*P*	*β*	*P*	*β*	*P*	*β*	*P*
Gender	−0.065	0.257	−0.074	0.182	−0.036	0.451	−0.052	0.275
Age	−0.125	0.035	−0.086	0.137	−0.057	0.254	−0.076	0.130
Marital status	0.139	0.015	0.145	0.008	0.080	0.099	0.082	0.084
Residence	−0.024	0.686	−0.003	0.964	−0.019	0.699	−0.018	0.714
Anemia	0.095	0.097	0.042	0.449	0.039	0.430	0.045	0.353
EMS								
Dummy_1	0.062	0.342	0.176	0.009	0.110	0.062	0.094	0.107
Dummy_2	−0.158	0.016	−0.016	0.818	−0.044	0.464	−0.046	0.440
DAS28-CRP			−0.308	< 0.001	−0.223	< 0.001	−0.218	< 0.001
Resilience					0.469	< 0.001	0.492	< 0.001
DAS28-CRP*Resilience							0.144	0.003
*F*	3.849		6.674		18.115		17.647	
Adjusted *R* ^*2*^	0.063		0.133		0.342		0.359	
*ΔR* ^*2*^			0.071		0.206		0.019	

Gender, women versus men; Marital status, married/cohabited versus single/divorced/widowed/separated; Residence, rural versus urban; Anemia, no versus yes; Dummy_1, EMS ≤ 1 versus no; Dummy_2, EMS > 1 versus no. * Indicated multiplication. Abbreviations: *MCS*, mental component summary; *EMS*, early morning stiffness; *DAS28-CRP*, Disease Activity Score 28-C-reactive protein

### Resilience as a mediator in the association between disease activity and MCS

With regard to the results of asymptotic and resampling strategies, disease activity was significantly and negatively associated with resilience (*β* = −0.181, *P* = 0.006). Because resilience was significantly and positively associated with MCS, a significant mediating role of resilience (effect size was −0.085, BCa 95% *CI*: −0.159, −0.028) in the association between disease activity and MCS was revealed. In addition, the proportion of mediating role of resilience was 27.6% in the total effect of disease activity on MCS.

## Discussion

In this study, both the PCS and MCS of HRQOL in patients with RA were significantly decreased compared to the general population of China [32]. This finding is consistent with previous studies conducted across countries, showing that all aspects of HRQOL of RA patients are impaired [3, 8, 33–35]. Also in agreement with most prior researches [7, 8, 10, 34–37], the score of the PCS was lower than the MCS, suggesting that RA has a greater impact on physical health than mental well-being. When compared with RA patients from other studies in Asian population, patients in our sample from mainland China tended to show a lower level of PCS [8, 12, 26, 36, 37]. The level of MCS was consistent with the results of the three studies mentioned above [8, 26, 37], but it was higher than the results from most previous studies across countries [7, 11, 12, 34–36, 38]. One important reason for the discrepancy in the MCS comparisons may be that considerable developments in treatment in recent years have had a significant beneficial effect on RA patients’ HRQOL [39]. However, for the discrepancy in the PCS comparisons, the disease can become very severe and difficult to control by the time rheumatological care is accessed due to limited knowledge or self-management about RA in mainland China [40]. Other reasons may be some differences in the sources and cultural backgrounds of subjects, and the interpretation of the concept of HRQOL across studies. Furthermore, patients with RA have significantly reduced levels of both PCS and MCS in comparison to those with other diseases, such as ankylosing spondylitis, psoriatic arthritis, and systemic lupus erythematosus [26, 35, 41]. In view of the above facts, policymakers and healthcare workers need to raise awareness of the improvement of HRQOL in patients with RA, and more resources should be made available in China.

In addition, we found that disease activity had significant impacts on physical and mental HRQOL. Our results were consistent with the most existing literatures strongly associating disease activity with the HRQOL of RA patients [7, 8]. This study extended the information available in the existing literature by showing that Chinese RA patients who experienced higher disease activity reported lower levels of HRQOL. RA patients in mainland China are prone to suffer from a higher disease activity due to the lack of early diagnosis, aggressive treatment and self-management [40]. In consequence, they were more likely to experience uncertainty concerning treatment, which could profoundly affect their HRQOL. The significant direct effect of disease activity on the HRQOL of RA patients emphasizes the importance of regular disease treatment and management. Based on the clinical evaluation of disease activity, a timely adjustment of drug treatment could reduce the degree of disease activity, and improve HRQOL in RA patients.

Resilience has been expected to predict and evaluate HRQOL among patients with chronic diseases [19–21, 24, 25]. Resilience was seldom studied in RA [18], a consensus was reached that higher level of resilience was beneficial for patients with RA and targeted psychological intervention required more emphasis. Consistent with previous studies [20, 21], resilience was strongly and positively associated with mental HRQOL in this study. RA patients with high resilience scores enjoyed high mental HRQOL, because they could recover easily and quickly from setbacks in their disease conditions. In general, resilience can allow RA patients to address their physical health correctly and to maintain a relatively good whole health state, resulting in a better HRQOL.

To our knowledge, the study was the first to explore the moderating role of resilience on the association between disease activity and HRQOL in RA patients. We found that disease activity and resilience interact with each other in their associations with mental HRQOL, but not physical HRQOL. In other words, resilience could attenuate the impact of disease activity on mental HRQOL. Moreover, psychosocial resilience should be hypothesized to moderate the association between extrinsic stressor and mental health according to Rutter’s protective mechanisms [17]. Also in other mental health domains, the interactions of resilience with extrinsic stressors have been confirmed. For instance, Ding et al. and Wingo et al. found that resilience moderated the association of childhood trauma with depressive symptoms [42, 43]. Resilience was found to be a protective factor for depressive symptoms and also mitigated the effects of peer victimization on depressive symptoms among rural-to-urban migrant children in China [44].

Some review literatures have indicated that resilience is a common response to physical disease diagnosis or treatment [45, 46]. The patients have an initial response that fits somewhere on the distress-resilience continuum; however, treatment or intervention experiences can modify the initial response through a process of recalibration. Johnston et al. has confirmed that resilience involves maintaining healthy levels of functioning following adversity and that it is a dynamic process not a personality trait. Therefore, in most previous studies, physical disease was either considered as an adversity leading to resilience or as a variable modifying the relationship between adversity and resilience. As a dynamic process, resilience varies across different disease stages [46]. Consistent with the above findings, we found that disease activity had a significantly negative impact on the level of resilience, indicating that poor physical condition could reduce the patient’s positive psychological capacities. Thus, the associations among disease activity, resilience, and mental HRQOL were in agreement with our hypothesis, disease activity also had an indirect effect on mental HRQOL through reducing resilience. This finding is in accordance with previous studies that showed the mediating roles of resilience in the relationships between cancer symptom distress and HRQOL, perceived symptoms and HRQOL in persons with long-term physical disabilities, and family function and HRQOL among the elderly [24, 25, 47, 48].

In view of these findings, as a target, resilience should be improved by effective positive psychological interventions in RA patients for coping with the negative effect of disease activity on HRQOL, especially mental well-being. Previous studies have developed some different strategies for resilience, such as cognitive behavioral and mindfulness-based interventions [49] and community-based meditative Tai Chi programme [50], and these interventions were effective in the context of HRQOL.

Several theoretical and practical strengths of this study should be highlighted. Theoretically, the study provided a preliminary evidence for the association between resilience and HRQOL, and the moderating and mediating roles of on the association between disease activity and HRQOL in Chinese RA patients. In practice, the low level of HRQOL in RA patients should be paid sufficient attention in China; in order to improve RA patients’ HRQOL, an integrated strategy should be implemented by using existing treatment and positive psychological intervention to reduce disease activity and develop resilience.

There are some limitations that should be illustrated in the present study. Firstly, due to the cross-sectional design of the study, it was unable to assess the causal relationships among study variables. It only provided a snapshot of the associations among disease activity, resilience and HRQOL in Chinese RA patients. Therefore, longitudinal study should be carried out further to verify our findings. Secondly, resilience and HRQOL were measured using self-administered questionnaires. The association between resilience and HRQOL might be influenced due to possible recall and reporting bias. Some effective process control measures have been carried out to minimize possible common-method bias. Thirdly, the present study was only conducted in a province of northeast China. Thus, the generalization of our findings requires to be further studied in other population with different cultural backgrounds. Finally, no differences in demographic characteristics and clinical variables were tested between participants and those who refused participation. The multi-center design and adequate sample size in this research could provide a good representation of patients with RA.

## Conclusions

Chinese RA patients experience impaired physical and mental HRQOL. Disease activity was negatively associated with both physical and mental HRQOL, and resilience was only positively associated with mental HRQOL. Resilience could mediate and attenuate the association between disease activity and mental HRQOL. In addition to controlling disease activity, targeted intervention strategies designed for resilience should be strengthened to improve the HRQOL of this population.

## Abbreviations

BCa 95% CI: Bias-corrected and accelerated 95% confidence intervals
DAS28-CRP: Disease Activity Score 28-C-reactive protein
EMS: Early morning stiffness
ERS: Ego-Resiliency Scale
HRQOL: Health-related quality of life
MCS: Mental component summary
PCS: Physical component summary
RA: Rheumatoid arthritis
SD: Standard deviation
SF-36: Medical Outcomes Study 36-item Short-Form Health Survey
SJC: Swollen joint counts
TJC: Tender joint counts
WCM: Wilson and Cleary model
